# Does early childhood education enhance parental school involvement in second grade?: Evidence from Midwest Child-Parent Center Program

**DOI:** 10.1016/j.childyouth.2020.105317

**Published:** 2020-10

**Authors:** Nishank Varshney, Sangyoo Lee, Judy A. Temple, Arthur J. Reynolds

**Affiliations:** a Humphrey School of Public Affairs, University of Minnesota, 301 19th Avenue South, Minneapolis, MN 55455, United States; b Human Capital Research Collaborative, University of Minnesota, 51 E River Road, Minneapolis, MN 55455, United States; c Institute of Child Development, University of Minnesota, 51 E River Road, Minneapolis, MN 55455, United States

**Keywords:** Parent involvement, Family engagement, Early childhood education, Preschool, Impact evaluation, Scale-up, CPC, Child-Parent Centers, MCPC, Midwest Child-Parent Centers, CLS, Chicago Longitudinal Study, MLS, Midwest Longitudinal Study, PI, Parent Involvement, MI, Multiple Imputation, IPW, Inverse probability weighting, ES, Effect Size, SD, Standard Deviation

## Abstract

This paper examines the impact on parent involvement in second grade in the Midwest Child-Parent Centers (MCPC), a high-quality preschool-to-third-grade school reform model. A new focus of research on early childhood programs is understanding how early childhood learning gains can be sustained. Two-generation programs that provide diverse family services may be one approach. The MCPC expansion was implemented for a cohort of over 2000 Chicago and Saint Paul students beginning in preschool. Based on a comparison of the program and usual-service comparison groups matched at the school level via propensity scores, ratings were obtained for a subset of the sample by teachers and parents on parent involvement in school in second grade. After accounting for potential attrition bias via multiple imputation and propensity score weighting, results indicated that MCPC participation was associated with significantly higher parent involvement in school at the end of second grade both in the aggregate sample (Effect Size = 0.19 SD) and in Chicago (ES = 0.24). Differences in Saint Paul, however, were small (ES = 0.15) and not statistically significant. Robustness testing using different model specifications revealed similar results. Implications for assessing and sustaining early childhood learning gains are discussed with a focus on recognizing that parental involvement is an integral component of high-quality programs.

## 1 Introduction

Parent involvement has been consistently recognized as a key program element that can promote the effectiveness of early childhood interventions (Castro et al., 2004, Joo et al., 2020, Reynolds and Temple, 2019, Zigler et al., 2006). The enduring effects on educational success and adult health reported by several landmark studies of early childhood intervention (Englund, White, Reynolds, Schweinhart, & Campbell, 2014) have been attributed by researchers to not only the fostering of early skills but also to the promotion by these interventions of increased parental support. Parent involvement can improve child outcomes by increasing learning time, enhancing child motivation and school commitment, and increasing expectations for achievement and success (Abenavoli, 2019, Reynolds and Temple, 2019). An important question asked in this study is whether a two-generation program offered to low-income urban families in preschool has an impact on school-based parental involvement several years later.

Parent involvement or family engagement in education has been of great interest to policymakers and consequently is mandated in various standards and legislative acts. In the federal preschool program Head Start, performance standards require that opportunities for involvement of parents or other family members must be offered (U.S. Department of Health and Human Services, 2016). In the No Child Left Behind (NCLB) Act of 2002, school districts receiving Title 1 funding were required to implement effective practices to promote parental involvement (Epstein, 2005). More recently, the Every Child Succeeds Act (ESSA) of 2015 continued to require school leaders to engage with families and imposed a requirement to devote a minimum of 1% of Title 1 funds to this purpose (Ross, 2016).

There are various dimensions to parental involvement (Epstein, 2010). The effects of parent involvement have been reported in various literature reviews and research syntheses. One strand of research focuses on which types of involvement are being promoted by preschool and home visiting programs, and the associations of this involvement with subsequent student academic success. Activities such as parents reading with their children, communications between parents and teachers, requirements that parents check and sign student homework, and parental expectations of student success are some of the types of parent involvement included in meta-analyses by researchers including Fan and Chen, 2001, Jeynes, 2012.

In a large review of the literature on the impact of parenting interventions on achievement and non-cognitive skills measured before the age of eight, Van Voorhis, Maier, Epstein, and Lloyd (2013) reported that the majority of studies from both non-experimental and experimental research designs demonstrate a positive link between involvement and student outcomes. In a meta-analysis of over 100 studies, Sheridan, Smith, Moorman Kim, Beretvas, and Park (2019) found that family-school interventions have a positive impact on children’s social-emotional outcomes. In another large meta-review of over 400 studies, Barger, Kim, Kuncel, and Pomerantz (2019) found small but significant effects of parents’ school-based involvement on children’s academic and non-academic adjustment. The findings from the literature are not entirely positive – for example, Fryer (2017) focused solely on Randomized Controlled Trials (RCT) intended to promote parental involvement with the goal of impacting student test scores and does not find strong results in general. His research (Fryer, Levitt, & List, 2015) involving an RCT providing financial incentives for certain parenting behaviors in an early childhood intervention showed mixed results on achievement – the achievement of white and Latino students improved but not that of black students.

The second strand of research that is more closely related to the goals of the current study focuses on whether parenting interventions can affect parenting behaviors. Analyzing the results from the Infant Health and Development Program (IHDP), Chaparro, Sojourner, and Huey (2019) reported that being offered a home visiting program followed by a high-quality preschool program for their child led to an increase in maternal care quality. In an evaluation of the Head Start Impact Study, which involved random assignment like the IHDP, Gelber and Isen (2013) found that parent involvement increased for families offered access to Head Start both during and after the intervention. Similarly, Puma, Bell, Cook, Heid, Broene, Jenkins, and Downer (2012) reported on the effects from the Head Start Impact Study and found evidence of improvements in parenting practices. In a non-experimental analysis of a large national sample, Bauer and Schanzenbach (2016) found that Head Start participation had an impact on positive parenting behaviors, especially for mothers without a high school degree. Recent studies continue to focus on preschool programs by examining the effects of randomly assigned Head Start enrichment programs intended to enhance parental involvement (Bierman, Welsh, Heinrichs, Nix, & Mathis, 2015).

## 2 Parent involvement and the Child-Parent Centers

The focus of the current study is on evaluating the effects of the Child-Parent Center (CPC) early education intervention. Originally implemented in public-school sites in Chicago in 1967, the CPC program offers a high-quality preschool through third-grade program emphasizing parental involvement to students and families in the city’s poorest neighborhoods. A long-running study called the Chicago Longitudinal Study (CLS) of a cohort of over 1500 students born in 1980 continues to follow these students into adulthood (Ou et al., 2020, Reynolds et al., 2018). The Child-Parent Center model regards parent involvement and engagement as one of its primary goals. It acknowledges the role of family members in supporting a child’s learning, achievement, and readiness for school (Hayakawa & Reynolds, 2016).

A number of studies in the CLS have reported associations between parental involvement and later school outcomes such as test scores and educational attainment (Barnard, 2004, Miedel and Reynolds, 1999). Hayakawa, Englund, Warner-Richter, and Reynolds (2013) elaborated on the importance of early parent involvement as an influential component in explaining continued achievement. They found that early parent involvement led to improved kindergarten achievement, which influenced children’s motivation in first grade. The higher motivated children in turn encouraged parents to continue their high levels of involvement throughout the elementary grades impacting later school achievement.

In a path analysis of the mechanisms through which CPC enhances well-being, family support behavior, including parent involvement in school, accounted for 26 percent of the total effect of preschool on high school completion and 22 percent on juvenile arrest (Reynolds, Ou, & Topitzes, 2004). These contributions generalize to elementary school achievement and adult outcomes ranging from reading achievement to mental health (Reynolds and Ou, 2011, Reynolds and Temple, 2019). In addition, the impact of early parent involvement on long term adult outcomes has been examined beyond academic performance. Research shows that early parent involvement had a mediating effect on lowering rates of substance abuse among adolescents (Hayakawa, Giovanelli, Englund, & Reynolds, 2016) and reduced the likelihood of ever smoking (Reynolds, Magro, Ou, & Eales, 2019).

## 3 Parent involvement in the Midwest expansion of the Child-Parent Centers

Starting in the year 2012, the CPC was expanded to several school districts in the Midwest as well as to more sites within Chicago as part of the federal funding initiative Investing in Innovation (i3). Some initial results of the preschool intervention on school readiness have been reported by Reynolds et al., 2014, Reynolds et al., 2016, Reynolds et al., 2016. The Midwest Child-Parent Center (MCPC) program assessed in this study is a preschool-to-third grade (P-3) school reform model. It was implemented in four school districts in the midwestern U.S.: Chicago, Evanston, and Normal in Illinois, and St. Paul in Minnesota. The program focuses on six core elements: collaborative leadership team, effective learning experiences, aligned curriculum and practices, parent involvement, professional development, and continuity and stability (Reynolds, Richardson, et al., 2016). While other elements are equally important to the program, the current study focuses on the parent involvement element of the model. Developed as a scalable program model, MCPC has a greater emphasis on collaborative leadership, continuity in instruction between grades, and menu-based parent involvement than earlier CPC models in the 1960–1990s (Reynolds, Hayakawa, et al., 2016). The Child-Parent Center program acknowledges the role of family members in supporting children’s learning, achievement, and readiness for school (Hayakawa & Reynolds, 2016). Every center of the MCPC program is equipped with a parent resource room for parents to interact with each other, meet with the program staff, or attend parent classes. These classes cover a wide range of topics such as GED/ESL (General Educational Development/English as a Second Language) preparation, nutritional education, learning financial skills, and employment training, among others. Parents participate in a needs assessment conducted at the beginning of the year to plan events that align with the identified needs of families in each center and meet their diverse needs. To promote parent participation, these activities are offered across different times of the day and week (Reynolds, Hayakawa, et al., 2016).

A menu-based system of parent involvement was developed through collaborations with principals, school staff, and the community, where family members identify which events are most beneficial for them to attend. Parents can choose from a range of activities in which to participate with expected participation of at least 2.5 h per week (Reynolds et al., 2017).

To manage the increased focus on parent involvement, each MCPC program requires a Parent Resource Teacher (PRT) and a School Community Representative (SCR) as core parent involvement staff members. The PRT is responsible for managing the parent resource room, communicating regularly with families, developing a monthly calendar of planned activities, implementing the identified workshops and activities, and maintaining a record of family participation in each activity through monthly logs. The SCR conducts home visits throughout the school year and is responsible for working with the families and community to identify resources available to help families meet their needs and support their children’s education.

Despite the importance of early parent involvement being well documented in the literature, there is limited research on how an early childhood program influences involvement not only in the preschool years but also in later years including the early elementary grades. If parents fail to continue their involvement past preschool, Stipek (2019) noted that the benefits of early engagement may fade out. In order to address this gap in the literature, we use detailed information on program participation and later parent involvement from the Midwest Child-Parent Center (MCPC) expansion project to investigate whether there is a sustained impact of a high-quality early childhood program on parent involvement as of second grade. Given that the detailed, later information on parental involvement was only collected for a subsample of the participants and controls, we employ rigorous non-experimental estimation methods to try to control for missing data on parent involvement. We investigate the impacts for the two school districts together and then separately by school districts. Our main research question is as follows: What is the impact of the MCPC program on parent involvement in 2nd grade?

## 4 Method

### 4.1 Participants and procedure

The CPC program was offered to more than 2500 preschool children across 26 schools from preschool through third grade in high-poverty neighborhoods in the selected school districts beginning in 2012. As a quasi-experimental longitudinal design, the Midwest Longitudinal Study (MLS) involved comparing students in CPC sites to comparison school sites using matching via propensity scores at the school level. Matched control sites were chosen based on the propensity score (which was estimated on key demographic characteristics of the schools including an ethnic breakdown and 3rd-grade test scores), the neighborhood of the school, and the schools’ willingness to participate in the study. A set of schools was created from these matched sites from which individual control students were selected and compared to the CPC students (Richardson, Reynolds, Temple, & Smerillo, 2017). Children in the comparison schools received half-day preschool programming which represents the usual publicly funded non-CPC preschool programming.

In this study, we limit our analysis to the two largest districts – Chicago, Illinois, and St. Paul, Minnesota which contained well-matched treatment and comparison groups. Due to local decisions made during the expansion of the CPC program to new cities, the Evanston and Normal implementations did not include non-treated comparison groups. As a requirement of project funding from the Investing in Innovation (i3) grants program of the U.S. Department of Education, the selection of treatment and comparison group sites, as well as the obtaining of consents for data collection for individual children in the study, was conducted by an outside evaluator (SRI, Inc.). A total of 3129 children participated in the study in both treatment and comparison school sites. Of that number, 2022 children participated in preschool at CPC program schools – 1724 in Chicago and 298 in St. Paul. Another 1107 children were followed as comparison group – 906 from Chicago and 201 from St. Paul. To be included in the analyses, all students had to meet the criteria of having been enrolled in preschool by January 2013 and stayed enrolled for at least 4 months.

A summary of the characteristics of the two groups is shown in Table 1. Children in the program group and comparison group were equivalent on child and family characteristics such as gender, single-parent family status, enrollment as 3-year-olds, and receipt of special education services. We account for the difference in some of the child and family risk characteristics between the intervention and the control group by including these as covariates in the main analysis. Importantly, pre-tests were administered to both treatment and control group members. In Chicago, across-group differences on three of the four measures were insignificantly different from zero while treatment group members scored higher on the literacy pre-test. In Saint Paul, only a literacy pre-test was administered and the findings show lower mean scores for CPC participants. These child and family characteristics will be included as covariates in the empirical analyses.

**Table 1:** Characteristics of CPC^a^ and comparison groups for Chicago and St. Paul Districts at Fall Baseline, 2012–2013.

Child/Family Characteristics^b^	Chicago			St. Paul		
	CPC (n = 1,724)	Comparison (n = 906)	Difference p-value^c^	CPC (n = 298)	Comparison (n = 201)	Difference p-value^c^
Female, %	51.6%	50.2%	0.512	52.5%	52.2%	0.950
Black, %	64.1%	45.6%	0.000	34.2%	27.4%	0.106
Hispanic, %	34.1%	53.8%	0.000	9.7%	13.4%	0.200
Asian/Pacific Islander, %	n/a	n/a	n/a	47.3%	41.8%	0.225
American Indian, %	n/a	n/a	n/a	3.7%	2.0%	0.276
White, %	n/a	n/a	n/a	5.0%	15.4%	0.000
Enrolled as three-year-old, %	40.4%	39.5%	0.649	n/a	n/a	n/a
Home language other than English, %	31.5%	55.8%	0.000	58.0%	45.2%	0.027
Special education status^d^, %	9.6%	9.2%	0.704	10.1%	5.5%	0.066
Child eligible for free or reduced lunch^e^, %	97.1%	94.6%	0.001	65.7%	48.3%	0.000
Mother completed high school	73.81%	65.30%	0.003	75%	86.15%	0.071
Single parent family status, %	42.43%	46.08%	0.244	31.08%	22.39%	0.192
Mother employed full- or part-time, %	47.09%	40.95%	0.053	63.01%	75.76%	0.068
Fall preschool score on Literacy, mean	35.65	32.81	0.003	6.94	10.63	0.000
Fall preschool score on Math, mean	23.63	23.74	0.835	n/a	n/a	n/a
Fall preschool Socioemot. devel., mean	40.91	41.47	0.502	n/a	n/a	n/a
Fall preschool score (total), mean	197.86	193.88	0.336	n/a	n/a	n/a

Note: a. CPC = Child-Parent Center. b. Data on child and family characteristics were collected from school administrative records and parent surveys. c. P values show the significance of mean (or percentage) group differences for the CPC and comparison groups. The comparison group participated in the Head Start and State PreK programs in Chicago and were matched on the school-level propensity to enroll in the program. d. Children have an Individualized Education Plan. e. Eligibility was defined at <130% of the federal poverty level.

At the beginning of the study, parents were asked for their consent to collect current and future information including assessments. Of the total 3129 kids originally in our sample, approximately 74% consented to share data (n = 2312), with the breakdown of 1783 from the program group (1571 Chicago; 212 St. Paul) and 529 from the comparison group (415 Chicago; 114 St. Paul). While the overall research project itself focused more on the determinants of schooling outcomes, in this paper we look more closely at the parent involvement outcomes that were obtained from all parents in the first year of the program for all the kids in the consented sample and approximately half of the sample during the fourth year of the program due to student mobility and other constraints. In the fourth year, detailed data on parent involvement were collected from 1093 participants – 845 from the program group (735 Chicago; 110 St. Paul) and 248 from the comparison group (198 Chicago; 50 St. Paul). Table 2 provides a summary of sample size and data loss over the years across the two districts. Our empirical strategy discussed in the next section involves different methods of controlling for missing outcomes.

**Table 2:** Sample size and attrition.

Sample	Chicago		St. Paul		Total		Grand Total
	CPC	Comparison	CPC	Comparison	CPC	Comparison	
Initial sample	1724	906	298	201	2022	1107	3129
Consented sample	1571	415	212	114	1783	529	2312
*Consent rate, %*	*91%*	*46%*	*71%*	*57%*	*88%*	*48%*	*74%*
Year 1 Parent Involvement	1571	415	212	114	1783	529	2312
Year 4 sample*	1311	363	221	79	1532	442	1974
Year 4 Parent Involvement	735	198	110	50	845	248	1093
*Year 4 response rate, %*	*56%*	*55%*	*50%*	*63%*	*55%*	*56%*	*55%*

Note: The sample size represents consented children who were still enrolled in the district’s schools in 2nd grade.

### 4.2 Measures of parent involvement

The original CPC program and the Midwest expansion focuses on strengthening the family-school relationship by actively inviting parents to participate in school settings.

Therefore, parent involvement in school is the focus of the study to measure the program's impact on parent involvement. Moreover, the CPC program highlights parent involvement not only in terms of parents engaging in cognitively stimulating behaviors with their children but also parents investing in themselves. Therefore, focusing on parents’ involvement in the school activities provided by the program captures a more holistic view of parent involvement and engagement. As part of MLS, data were collected from multiple sources to measure the extent of parent involvement in school through the years starting in 2012. Baseline data for the first year of enrollment (2012–13) were collected in preschool. Reynolds et al., 2016, Reynolds et al., 2016 report positive effects associated with the CPC program on parent involvement at the end of preschool. Data from the fourth year of the study (2015–16), i.e. second grade, are the focus of the current research. While in the first year of the study (2012–13), a survey was administered to the parents, in the fourth year of the study (2015–16), a survey was administered to the teachers. Data on the involvement of parents in their child’s school activities was collected using four sources described below.

1. *Parent Involvement Log Data* - During both the study years (2012–13 and 2015–16), teachers maintained a log of individual parents’ involvement in the school activities, which was collected and analyzed for this study. The obtained measure was a continuous variable of the number of events attended by a child’s parents in the given year. This measure was then coded into a categorical variable, considering a school year of 9 months, to align with the other parent involvement measures as following: If the parents attended no events during the school year it was coded as ‘never’; participation in 1–6 events was coded as ‘less than once a month’; 7–10 events was coded as ‘once a month’; 11–18 events was coded as ‘2–3 times a month’; 19–35 events was coded as ‘once a week’, and attendance at more than 36 events was coded as ‘more than once a week.’
2. *Parent Survey -* During the first year of the study (2012–13), parents were administered a survey and asked the question: “So far this year, frequency participated in school or center activities”, among others. The question had six response options – Never, less than once a month, once a month, 2–3 times a month, once a week, and more than once a week. Values of 1 (never) through 6 (more than once a week) were assigned to create a measure of parent involvement using parent survey.
3. *Teacher Checklist -* During the fourth school year of the study (2015–16), teachers across the program and comparison sites were asked 13 questions related to the parents’ involvement in various activities at the school (see Appendix A for the list of questions). The checklist had the same six response options as those in the parent survey from the first year. We calculated the mean value of responses for the 14 questions and recoded them into the same six categories (by rounding to the nearest integer) to create a measure for parent involvement using the teacher checklist.
4. *Teacher Survey -* During the fourth school year of the study (2015–16), the teachers were asked to report the percentage of parents attending school activities or events across the same six-point scale as used in the parent survey and teacher checklist. We then calculated the weighted mean of this measure and recoded it into the six-point scale (by rounding to the nearest integer) to calculate the average parent involvement at the classroom level.

For example, if a teacher reported the percentage of parents attending school activities or events as follows: Never – 15%, less than once a month – 10%, once a month – 15%, 2–3 times a month – 20%, once a week – 35%, and more than once a week – 5%. We calculated the weighted mean as 1∗0.15+2∗0.1+3∗0.15+4∗0.20+5∗0.35+6∗0.05=3.65*.* We then rounded this measure to the nearest integer, in this case, 4. We then allotted this score to all the children in this classroom, which corresponds to participating ‘2–3 times a month’, on average.

Previous studies show that the teachers’ ratings of parent involvement are valid measures of their participation in school activities (Barnard, 2004). Using classroom level data can help reduce response bias and ‘halo’ effects that can be found in ratings of individual children (Reynolds, Richardson, et al., 2016). Castro et al. (2004) found that the teachers’ reports of parent involvement had a higher correlation with the log data than the parents’ self-reports. Other studies of the Child-Parent Centers have used self-reported data from parent surveys (Reynolds et al., 2014) and the log data to measure parent involvement (Reynolds et al., 2017). Measures that combine parent and teacher reports also have been used (Reynolds, 2000) and likely tap a wide range of participation events, thus enhancing content and construct validity. We next describe the composite measures of parent involvement from year 1 (the end of preschool) and year 4 (the end of second grade).

*First-year composite measure* of parent involvement was created using the data from the teacher’s log of the parent’s school involvement activities and the parent survey. We use the teacher’s log of parent involvement as the primary measure, but when information is missing the parent survey is used instead. As shown in Table 3, the mean composite rating on the six-point scale for the program and comparison groups in Chicago was 3.2 and 1.8, respectively (Total mean: 2.88; SD = 1.70). For St. Paul, the corresponding ratings were 2.4 and 1.9, respectively (Total mean: 2.21; SD = 1.12). A rating of 3 indicates participation in school two to three times per month whereas a rating of 2 corresponds to participating in one event a month.

**Table 3:** Mean values of parent involvement for different data sources with the sample size.

Data Source	Chicago		St. Paul	
	CPC	Comparison	CPC	Comparison
**Year 1 (Pre-K)**				
Parent Log Data	3.2 (n = 1570)	1.8 (n = 415)	2.4 (n = 212)	1.9 (n = 114)
Parent Survey	3.4 (n = 1169)	3.2 (n = 317)	2.6 (n = 144)	2.5 (n = 68)
Composite Measure*	*3.2* *(n = 1571)*	*1.8* *(n = 415)*	*2.4* *(n = 212)*	*1.9* *(n = 114)*
**Year 4 (2nd grade)**				
Parent Log Data	2.7 (n = 294)	2.0 (n = 1)	2.0 (n = 46)	2.5 (n = 2)
Teacher Survey	2.4 (n = 307)	2.8 (n = 117)	2.0 (n = 65)	2.2 (n = 24)
Teacher Checklist	1.9 (n = 559)	2.0 (n = 182)	1.6 (n = 103)	1.6 (n = 47)
Composite Measure*	*2.2* *(n = 735)*	*2.0* *(n = 198)*	*1.7* *(n = 110)*	*1.7* *(n = 50)*

Note: Each parent involvement data source category ranges from 1 to 6. Detailed measure descriptions are in Table 6. The composite measure for each of the two years was created using the data sources available for the corresponding year. 2nd grade PI Composite measure (Sample Size – Chicago: 933; St. Paul: 160) is used as the dependent variable in all further analysis.

*Fourth-year composite measure* of parent involvement was created using the data from the teacher’s log of parent involvement, the teacher survey, and the teacher checklist. We first assigned the value of parent involvement from the teacher’s log as a composite variable and then filled in the missing values using teacher checklist data for the cases where the log data was unavailable. If data from both these sources were unavailable, then we filled in the missing values using the data from the teacher survey. As shown in Table 3, the mean composite ratings across these sources for the Chicago program and comparison groups were 2.2 and 2.0, respectively (Total mean: 2.16; SD = 1.06). For St. Paul, the respective values were 1.7 and 1.7 (Total mean: 1.73; SD = 0.55). For both the districts combined, the average values for parent involvement composite measure in the second grade were 2.1 and 2.0 respectively (Total mean: 2.10; SD = 1.02). On average, these ratings show a decline in parent involvement in second grade with an average participation of about once per month.

Table 3 shows the mean of parent involvement measures for each of the different data sources for both the districts. With the many sources of data utilized, the composite ratings are inclusive of a wide range of parental school involvement activities. The ratings revealed some differences by source. In year 1 (preschool), parent survey reports showed a higher level of parent involvement, which was nearly double the rating of log data documenting participation records at the school. In year 4 (2nd grade), the teacher survey at the classroom level and parent log data showed similar mean ratings but the teacher checklist data on individual students showed substantially lower mean ratings. Although teachers have substantial knowledge about parental school involvement, they may not be fully aware of all types of participation for their students. Hence, a composite measure may provide a more valid profile of involvement.

More details about the parental involvement measures are shown in Table 4a, Table 4b. While our estimation focuses on the factors predicting parent involvement in year 4 (by the end of second grade), we again report information from both the preschool year as well as 4 years later. Looking at these data shown as involvement by month or even more frequently, the results suggest that parent involvement was higher overall in the preschool years and few parents continued their involvement on a weekly or more often basis as their children advanced in school. The impact of the MCPC program on parent involvement at the end of preschool was analyzed by Reynolds et al., 2014, Reynolds et al., 2016, Reynolds et al., 2016.

**Table 4a:** Frequency table of children meeting each parent involvement composite variable category – Chicago.

Category	Year 1 Parent Involvement Composite Measure			Year 4 Parent Involvement Composite Measure		
	CPC (n = 1722)	Comparison (n = 906)	Total (n = 2,628)	CPC (n = 740)	Comparison (n = 199)	Total (n = 939)
Never, %	16.67	76.60	37.33	21.49	26.63	22.58
Less than once a month, %	38.33	15.34	30.40	56.62	54.77	56.23
Once a month, %	7.90	4.97	6.89	10.41	11.06	10.54
2–3 times a month, %	9.35	2.98	7.15	5.27	5.53	5.32
Once a week, %	12.95	0.11	8.52	4.05	1.01	3.41
More than once a week, %	14.81	0.00	9.70	2.16	1.01	1.92
Total, %	100	100	100	100	100	100

**Table 4b:** Frequency table of children meeting each parent involvement composite variable category – St. Paul.

Category	Year 1 Parent Involvement Composite Measure			Year 4 Parent Involvement Composite Measure		
	CPC (n = 212)	Comparison (n = 115)	Total (n = 327)	CPC (n = 110)	Comparison (n = 50)	Total (n = 160)
Never, %	16.51	14.78	15.90	28.18	36.00	30.63
Less than once a month, %	61.79	85.22	70.03	70.91	58.00	66.88
Once a month, %	5.66	0.00	3.67	0.00	4.00	1.25
2–3 times a month, %	4.72	0.00	3.06	0.91	2.00	1.25
Once a week, %	4.25	0.00	2.75	0.00	0.00	0.00
More than once a week, %	7.08	0.00	4.59	0.00	0.00	0.00
Total, %	100	100	100	100	100	100

Some changes in parent involvement in the school setting are evident over time and across the two districts. For both Chicago and St. Paul district, parent involvement in school setting decreases from preschool to second grade. Besides, one can observe that for both CPC and comparison sites, parents are more likely to be involved in school settings during preschool, and the involvement decreases by second grade. The difference, however, is that parents with children who attended CPC sites are more likely to be involved than parents with children who attended comparison sites despite the overall decreasing involvement trend over the years. Parents who participated in any parent involvement events in the school setting were 78.5% (CPC) vs 73.4% (Comparison) for the Chicago district and 71.8% (CPC) vs 64.0% (Comparison) the St. Paul district.

In the next section, we focus our attention on estimating the effects of participation in the CPC program on parent involvement at the end of second grade. The greatest empirical challenge is the amount of missing data on the outcome of interest. Table 5 provides a summary of the family and child characteristics of the children that had data on second-grade parent involvement and those that did not. Data on some of the child and family risk measures were missing if the parents chose not to respond to those questions in the parent survey (see Appendix B). There was no evidence that the probability of missing the key outcome of interest varied across treatment and comparison groups in either Chicago or Saint Paul nor by gender or maternal employment. The estimation methods described below attempt to control for the differences across observed characteristics in the probability of having a missing outcome on the outcome of interest.

**Table 5:** Mean values of explanatory variables for students with and without 2nd grade parent involvement (PI) information.

Child/Family characteristics^a^	Chicago			St. Paul		
	Missing PI (n = 1,053)	Non-Missing PI (n = 933)	Difference p-value^b^	Missing PI (n = 166)	Non-Missing PI (n = 160)	Difference p-value^b^
Any CPC Preschool, %	79.39	78.78	0.737	61.45	68.75	0.168
Female, %	51.38	52.73	0.546	50.91	48.75	0.675
Black, %	68.38	58.63	0.000	41.57	26.25	0.003
Hispanic, %	30.96	40.51	0.000	10.84	15.00	0.264
Asian/Pacific Islander, %	n/a	n/a	n/a	31.93	46.88	0.006
American Indian, %	n/a	n/a	n/a	3.61	4.38	0.727
White, %	n/a	n/a	n/a	12.05	7.50	0.169
Home language other than English, %	28.11	38.05	0.000	46.39	60.63	0.010
Special education status^c^, %	8.08	7.50	0.632	12.65	11.88	0.832
Child eligible for free or reduced lunch^d^, %	97.82	98.61	0.188	85.54	93.13	0.027
Mother completed high school, %	75.45	69.17	0.008	77.23	71.88	0.332
Single parent family status, %	47.36	42.10	0.038	33.98	27.00	0.222
Mother employed full- or part-time, %	46.27	46.00	0.913	67.05	61.94	0.442
Fall preschool score on Literacy, mean	32.17	36.96	0.000	10.46	7.57	0.014
Fall preschool score on Math, mean	22.36	24.77	0.000	n/a	n/a	n/a
Fall preschool Socioemot. devel., mean	39.20	43.66	0.000	n/a	n/a	n/a
Fall preschool score total, mean	186.82	206.81	0.000	n/a	n/a	n/a

Note: a. Data on child and family characteristics were collected from school administrative records and parent surveys. b. P values show the significance of mean (or percentage) group differences for the children for whom parent involvement data in 2nd grade was available and the children for whom this data was missing. c. Children have an Individualized Education Plan. d. Eligibility was defined at < 130% of the federal poverty level.

## 5 Results

Our empirical strategy involves the estimation of several linear models with different approaches used to handle missing explanatory variables and missing parental involvement outcomes. All regression models control for the influence of the following covariates listed in Table 5: fall baseline performance in early literacy, child's gender, race/ethnicity, immigrant/refugee status, special education assignment in pre-school, household language other than English, eligibility for subsidized lunches, whether the child lived with a single parent, mother’s education status, mother’s employment status, whether the household income was within 185% of the federal poverty level, whether the parents moved during the last one year, and a dichotomous indicator of whether parents received any government assistance during the last year. These covariates were measured at the fall baseline through parent surveys and school administrative records.

To account for missing data, we use two methods of data imputation – simple imputation and Multiple Imputation (MI). In the first method, we first fill in missing explanatory variables with the mean of continuous measures calculated from the students not missing this information. For categorical variables, we fill in using the mode. Further, we include a series of missing data indicator variables in the empirical analysis.

In the second method, using the *mi estimate* command in Stata 15, we obtain estimates of regression coefficients by creating several imputed data sets with imputations drawn from an underlying distribution of the observed data to estimate multiple values that reflect the uncertainty around the true value of the missing explanatory variables. In these initial analyses, both the simple imputation and the multiple imputation approaches are conducted only for the sample of 1093 students who have data on the parent involvement outcome. As explained by Donders, van der Heijden, Stijnen, and Moons (2006), a benefit of using multiple imputation is that MI can result in obtaining correctly estimated standard errors while simple imputation results in estimated standard errors that are too small. Stata’s multiple imputation estimator is suitable for two different assumptions regarding the missing data: data missing completely at random (MCAR) and missing at random (MAR). Missing at random refers to the possibility that the students’ probability of having missing data is correlated with the included covariates but the actual values of the missing data are not.

Two additional methods make use of the larger analytic sample consisting of students who are lacking the key outcome of interest. One method involves the use of Inverse Propensity Score weighting (IPW) and the final method involves using MI to simulate regression estimates for both the missing explanatory variables as well as the independent variable. Our analytic strategy involves estimating four regression models that take different approaches to address missing data. In addition to using imputation for missing data, we address concerns about missing outcomes through the use of Inverse Propensity Weighting (IPW) and also through a combination of IPW and multiple imputation. While IPW is more often used when treatment assignment is non-random, it can also be of use in attrition analyses (Seaman & White, 2013). Data were analyzed in Stata, Version 15 (StataCorp, 2017).

The IPW results make use of the larger analytic sample to better understand and control for the differences in characteristics of students who have and who are missing parent involvement measures at the end of second grade. To do this, we first run a probit regression using the larger sample of students with and without parent involvement information in second grade to generate predicted probabilities for each student of having this information using observable characteristics assumed to influence attrition. Being able to predict missingness for the dependent variable is important. Hence the prediction model includes a comprehensive set of child and family risk variables that also will be included in the main outcomes regressions as well as a set of additional variables (see Appendix C).

With the results of the probit estimation, we then assign a weight of 1/p1 to each child, where p1 is the predicted probability of the child being in the recovery sample (Reynolds, Temple, Ou, Arteaga, & White, 2011). While only the students having data on the parental involvement outcome are included in the weighted regression, there exist significant differences in their probability of reporting the second-grade outcome. Students with characteristics associated with a lower predicted probability of being included in the analysis (but who actually have information for the second-grade outcome) are rarer and consequently are given larger weight. Students with characteristics associated with a higher predicted probability of being in the regression are assigned smaller weights. In a sense, the use of IPW reweights the sample to better resemble a sample in which the outcome is missing at random. Our approach in Model 3 has been used by other researchers using longitudinal data, for example in Caldwell et al. (2008), who used IPW to adjust for the fact that over 40% of the outcome of interest was missing in a longitudinal follow-up of survey participants who also had missing data on explanatory variables addressed using MI. Additional possibilities for combining IPW and MI are discussed in Seaman, White, Copas, and Li (2012).

Table 6, Table 7, Table 8 report regression results from Chicago, Saint Paul, and the two districts aggregated, respectively. Models 1 and 2 can be compared to examine the results arising from simple imputation and multiple imputation for missing explanatory variables. In Model 3, multiple imputation for the explanatory variables is combined with Inverse Propensity Score weighting to reweight the observed sample of students with data available on the parent involvement outcome. For Chicago, the sample size used in the initial regression models is 933 students while in Saint Paul the sample size is 160. Finally, to better understand the robustness of the methods we employ, in Model 4, multiple imputation procedure is applied to the explanatory variables as the outcome variable. Model 4 results are reported for the larger sample of 1963 students in Chicago and 326 students in Saint Paul.

**Table 6:** Estimation results: Effects of CPC preschool participation on second grade parent involvement for Chicago District.

Variables	**Model 1** Simple Imputation^2^ (Indep Vars only)	**Model 2** Multiple Imputation (Indep Vars only)	**Model 3** MI & IPW (Indep Vars only)	**Model 4** Multiple Imputation (Both Dep and Indep Vars)
CPC preschool participation	0.208*	0.231**	0.251**	0.199**
	(0.106)	(0.112)	(0.117)	(0.0884)
Fall baseline score	−0.00248	−0.00114	−0.00125	−0.00195**
	(0.00158)	(0.000962)	(0.000968)	(0.000822)
Single parent	−0.0315	−0.0578	−0.0524	−0.0950
	(0.127)	(0.0978)	(0.0907)	(0.101)
Mother employed	−0.153**	−0.144*	−0.136*	−0.117
	(0.0744)	(0.0771)	(0.0774)	(0.0758)
Mother high school graduate or above	−0.00904	0.00206	0.0109	0.0331
	(0.112)	(0.0994)	(0.101)	(0.1000)
Child eligible for free or reduced lunch	−0.0916	−0.156	−0.149	−0.249
	(0.245)	(0.235)	(0.237)	(0.364)
HH income within 185% of federal poverty	−0.131	−0.107	−0.110	−0.187*
	(0.139)	(0.127)	(0.128)	(0.106)
Family moved in last 12 months	−0.162**	−0.127*	−0.131*	−0.183*
	(0.0680)	(0.0668)	(0.0658)	(0.0925)
Family receives any government assistance	−0.420**	−0.327**	−0.332**	−0.301*
	(0.158)	(0.141)	(0.149)	(0.170)
Hispanic	0.212*	0.265*	0.276*	0.201
	(0.121)	(0.147)	(0.148)	(0.237)
Black	0.254*	0.339**	0.353**	0.330
	(0.128)	(0.141)	(0.143)	(0.234)
Female	0.0922*	0.0725	0.0690	0.0658
	(0.0455)	(0.0471)	(0.0480)	(0.0652)
Family belongs to immigrant/refugee group	−0.304**	−0.251*	−0.264*	−0.211
	(0.137)	(0.136)	(0.135)	(0.139)
Special education	−0.102	−0.141	−0.146	−0.211
	(0.141)	(0.133)	(0.135)	(0.182)
Household native language not English	0.312*	0.335*	0.341*	0.396***
	(0.160)	(0.172)	(0.170)	(0.114)
Constant	3.016***	2.429***	2.411***	2.779***
	(0.468)	(0.328)	(0.320)	(0.481)
Observations	933	933	933	1986
R-squared	0.077			

Notes: 1. In Model 1, missing data on explanatory variables are filled in with the mean value and a set of missing data indicators are included but not shown. The estimated coefficients on the missing data indicators are not significantly different from zero. 2. The effect sizes of CPC program participation on second-grade parent involvement outcome for the four models are 0.20, 0.22, 0.24, and 0.19 respectively. 3. Standard errors clustered at the preschool site in parentheses. 4. Statistical Significance Levels ***p < 0.01, **p < 0.05, *p < 0.1.

**Table 7:** Estimation results: Effects of CPC preschool participation on second grade parent involvement for St. Paul District.

Variables	**Model 1** Simple Imputation^2^ (Indep Vars only)	**Model 2** Multiple Imputation (Indep Vars only)	**Model 3** MI & IPW (Indep Vars only)	**Model 4** Multiple Imputation (Both Dep and Indep Vars)
CPC preschool participation	0.0806	0.0751	0.0835	0.120
	(0.122)	(0.126)	(0.130)	(0.0840)
Upper Alphabet Fall Baseline Score	−0.00901*	−0.00626	−0.00652	−0.00788
	(0.00483)	(0.00459)	(0.00477)	(0.00454)
Single parent	−0.00340	0.0295	0.0166	0.0446
	(0.0666)	(0.111)	(0.106)	(0.120)
Mother employed	0.0161	0.0175	0.0147	−0.0478
	(0.102)	(0.129)	(0.127)	(0.117)
Mother high school graduate or above	0.362**	0.162	0.177	0.279**
	(0.122)	(0.124)	(0.122)	(0.119)
Child eligible for free or reduced lunch	0.0316	−0.0835	−0.0275	0.0849
	(0.126)	(0.172)	(0.175)	(0.205)
HH income within 185% of federal poverty	−0.179*	−0.127	−0.132	−0.145
	(0.0975)	(0.126)	(0.122)	(0.163)
Family moved in last 12 months	−0.0179	−0.0223	−0.0162	−0.0709
	(0.111)	(0.115)	(0.109)	(0.106)
Family receives any government assistance	−0.335***	−0.166	−0.226*	−0.483**
	(0.0834)	(0.125)	(0.117)	(0.173)
Asian/Pacific Islander (rel. to Caucasian/White)	0.0332	0.0429	0.0291	0.140
	(0.182)	(0.207)	(0.211)	(0.160)
Hispanic	0.0932	0.0767	0.0669	0.136
	(0.208)	(0.237)	(0.231)	(0.187)
African American	−0.224	−0.222	−0.231	−0.143
	(0.184)	(0.203)	(0.205)	(0.162)
American Indian	−0.177	−0.0514	−0.0534	−0.0558
	(0.137)	(0.257)	(0.255)	(0.264)
Female	−0.0396	−0.0394	−0.0390	−0.0444
	(0.0687)	(0.0668)	(0.0600)	(0.0643)
Family belongs to immigrant/refugee group	0.194	0.0832	0.112	0.147
	(0.141)	(0.135)	(0.132)	(0.151)
Special education	−0.114	−0.0899	−0.0822	−0.0110
	(0.137)	(0.150)	(0.154)	(0.140)
Household native language not English	0.0556	0.0761	0.0599	−0.0201
	(0.0463)	(0.0755)	(0.0741)	(0.148)
Constant	1.776***	1.889***	1.885***	1.908***
	(0.214)	(0.238)	(0.234)	(0.254)
Observations	160	160	160	326
R-squared	0.193			

Notes: 1. In Model 1, missing data on explanatory variables are filled in with the mean value and a set of missing data indicators are included but not shown. The estimated coefficients on the missing data indicators are not significantly different from zero. 2. The effect sizes of CPC program participation on second-grade parent involvement outcome for the four models are 0.15, 0.14, 0.15, and 0.22 respectively. 3. Standard errors clustered at the preschool site in parentheses. 4. Statistical Significance Levels ***p < 0.01, **p < 0.05, * p < 0.1.

**Table 8:** Estimation results: Effects of CPC preschool participation on second grade parent involvement for Chicago and St. Paul Districts Combined.

Variables	**Model 1** Simple Imputation^2^ (Indep Vars only)	**Model 2** Multiple Imputation (Indep Vars only)	**Model 3** MI & IPW (Indep Vars only)	**Model 4** Multiple Imputation (Both Dep and Indep Vars)
CPC preschool participation	0.192**	0.184**	0.198**	0.138**
	(0.0903)	(0.0887)	(0.0908)	(0.0684)
Standardized Fall Baseline Test Score	−0.398	−0.242	−0.258	−0.393***
	(0.276)	(0.212)	(0.219)	(0.133)
Single parent	−0.0140	−0.0359	−0.0336	−0.0362
	(0.109)	(0.0849)	(0.0809)	(0.0748)
Mother employed	−0.145**	−0.109*	−0.105	−0.0952
	(0.0668)	(0.0617)	(0.0633)	(0.0646)
Mother high school graduate or above	0.0192	0.0127	0.0235	0.0246
	(0.103)	(0.0920)	(0.0942)	(0.0772)
Child eligible for free or reduced lunch	−0.0712	−0.0869	−0.0931	−0.131
	(0.167)	(0.175)	(0.179)	(0.236)
HH income within 185% of federal poverty	−0.170	−0.102	−0.0996	−0.194
	(0.122)	(0.109)	(0.108)	(0.132)
Family moved in last 12 months	−0.130*	−0.108	−0.108	−0.146**
	(0.0661)	(0.0692)	(0.0690)	(0.0592)
Family receives any government assistance	−0.394***	−0.301**	−0.307**	−0.358***
	(0.140)	(0.127)	(0.131)	(0.106)
Asian/Pacific Islander (rel. to Caucasian/White)	−0.00571	−0.0973	−0.100	−0.0194
	(0.214)	(0.227)	(0.218)	(0.275)
Hispanic	−0.125	−0.144	−0.147	−0.101
	(0.238)	(0.235)	(0.228)	(0.266)
African American	−0.0905	−0.116	−0.126	−0.0625
	(0.207)	(0.193)	(0.184)	(0.227)
American Indian	−0.0984	−0.0640	−0.0756	0.0881
	(0.283)	(0.259)	(0.254)	(0.370)
Hispanic & African American	−0.0262	−0.0664	−0.0566	0.0983
	(0.324)	(0.293)	(0.292)	(0.306)
Female	0.0715*	0.0639	0.0647	0.0656
	(0.0397)	(0.0403)	(0.0416)	(0.0520)
Family belongs to immigrant/refugee group	−0.220**	−0.127	−0.123	−0.137
	(0.0881)	(0.0869)	(0.0866)	(0.113)
Special education	−0.0955	−0.117	−0.127	−0.139
	(0.108)	(0.116)	(0.117)	(0.127)
Household native language not English	0.283**	0.276*	0.269*	0.307*
	(0.126)	(0.142)	(0.139)	(0.153)
Chicago School District	0.569***	0.517***	0.519***	0.530***
	(0.130)	(0.130)	(0.130)	(0.0930)
Constant	2.353***	2.151***	2.152***	2.353***
	(0.286)	(0.252)	(0.248)	(0.233)
Observations	1093	1093	1093	2312
R-squared	0.085			

Notes: 1. In Model 1, missing data on explanatory variables are filled in with the mean value and a set of missing data indicators are included but not shown. The estimated coefficients on the missing data indicators are not significantly different from zero. 2. The effect sizes of CPC program participation on second-grade parent involvement outcome for the four models are 0.19, 0.18, 0.19, and 0.14 respectively. 3. Standard errors clustered at the preschool site in parentheses. 4. Statistical Significance Levels ***p < 0.01, **p < 0.05, *p < 0.1.

Model 3 (IPW/MI) represents our preferred specification. Multiple imputation is used when the individuals are missing values for the explanatory variables and this imputation also is incorporated in the probit model that predicts whether or not students have second-grade parent involvement information. But given the significance of the data loss on the dependent variable, we heed Seaman et al. (2012)’s caution that inadequacies in the imputation model could be of greater concern and lead to considerable bias. Instead of imputing the outcome, Model 3 combines IPW to reweight the set of cases that have information on the outcome so that the resulting sample is more representative of the larger sample.

A comparison of the results generated from these different methods for addressing missing data is discussed below where we begin our analyses of examining the impact of preschool CPC participation on second-grade parent involvement in the school setting for Chicago and St. Paul districts separately.

### 5.1 Chicago district

Table 6 shows results from regression analyses of the association between preschool CPC participation on second-grade parent involvement for the students who attended the Chicago Public Schools. The column labeled Model 1 contains estimates from a standard linear regression in which participation in the CPC program is the key policy variable of interest included along with a set of covariates such as the early literacy pre-test score and child and family demographic characteristics. Missing data for these characteristics are replaced by the median or mode and additional indicator variables reflecting whether or not each variable as imputed is included in the regression but not shown in the table. CPC program attendance is significantly associated with higher levels of parent involvement in school at the end of second grade. The estimated coefficient of 0.208 on CPC participation corresponds to a 0.20 Standard Deviation (SD) increase in second-grade parent involvement. Next, we use a multiple imputation approach to fill missing values of explanatory variables whose result is shown in Model 2. Using this model, CPC attendance during preschool year again is associated with significantly higher second-grade parent involvement by 0.22 SD. As predicted, Model 1 contains a larger set of estimated coefficients that have achieved statistical significance as compared to Model 2.

But to what extent are the results in Models 1 and 2 affected by the significant reductions in sample size arising from the lack of missing data on the outcome? Both Models 3 and 4 make use of the information from the larger sample by including in the analyses those students who lack parent involvement information in second grade. In addition to using multiple imputation for the missing covariates, we apply Inverse Probability Weighting (IPW) to account for program attrition from preschool to second grade as described in the Methods section above. This weighted regression (Model 3) shows that CPC program attendance in preschool is associated with significantly higher second-grade parent involvement by 0.24 SD. Finally, we apply the multiple imputation approach to fill missing values on both - the explanatory as well as the outcome measures. As shown in model 4, CPC attendance in preschool is significantly associated with a 0.19 SD increase in second-grade parent involvement.

Overall, the Chicago district’s analysis indicates that participation in the CPC program improves the rate of parent involvement in second grade compared to children who attended a regular preschool program in similar socio-demographically matched sites. Results do not seem sensitive to the method used to control for missing data.

### 5.2 St. Paul district

Following the same model application from the Chicago analyses but using a much smaller sample, results for St. Paul district are reported in Table 7. Model 1 shows standard linear regression with the simple imputation approach where CPC program attendance is associated with a 0.15 SD increase in second-grade parent involvement; however, the difference is statistically insignificant. In Model 2, using MI on explanatory variables, CPC program attendance in the preschool year is associated with a 0.14 SD increase in parent involvement in second grade. However, this difference is also not statistically significant at the 10% level. Next, we combine IPW with MI in model 3, where CPC program participation in preschool is associated with a 0.15 SD increase in parent involvement in second grade. Finally, applying multiple imputation on both - explanatory and outcome measures in model 4, we observe that the CPC attendance in preschool is associated with a 0.22 SD increase in second-grade parent involvement. As with the other models, these estimates of the effects of the CPC program are not significantly different from zero at the 10% level of significance. Given the rigorous approach to deal with missing data from program attrition, we believe that our findings are robust. However, due to a much smaller sample size in the St.Paul district, there is a possibility that the statistical power may be weak.

### 5.3 Chicago and St. Paul districts combined

During the implementation period of the CPC program, each school was assessed on the program implementation quality using a rubric created by the researchers based on program guidelines. Implementation fidelity scores reflect how well the CPC program was implemented at each of the program sites. We analyzed the impact of CPC participation on second-grade parent involvement separately for the Chicago and St. Paul districts as the two districts had different implementation fidelity scores as shown in Appendix D. However, to estimate the impact of the CPC program as a scale-up intervention program, in Table 8, we show the results for both the districts combined. In the combined regression, the early literacy pretests, which were different assessments in the two districts, are normalized to allow better comparisons.

Applying the same methodological approach from the two districts above, we also introduce a control for the district variable to capture any variation coming from different districts. The simple imputation model (Model 1) shows that CPC participation in preschool is associated with a statistically significant increase of 0.19 SD parent involvement in second grade. The MI model on explanatory variables (Model 2) shows that program participation is associated with a 0.18 SD increase in parent involvement in second grade which is also significant at 5% level. In the IPW and MI combined analysis (Model 3) we observe that CPC attendance is significantly associated with a 0.19 SD increase in second-grade parent involvement. In model 4, employing MI on both dependent and independent variables, program participation is associated with a statistically significant 0.14 SD increase in second-grade parent involvement.

## 6 Discussion

In this study, we follow a cohort of students who attended publicly funded preschool programs in high-poverty neighborhoods of urban school districts in St. Paul, MN, and Chicago, IL. Making use of the detailed parent involvement information collected on a subset of preschool participants and their families, we employ multiple imputation and inverse probability weighting approaches to address missing data concerns. Our results indicate that participation in preschool programs emphasizing the role of parents in children’s early schooling is associated with higher parent involvement with effect sizes ranging from 0.14 to 0.19 depending on the model specification.

Our study contributes to the literature in multiple ways. This is the first evaluation study looking at the impact of the Midwest Expansion of the Child-Parent Center's early childhood intervention on parent involvement in second grade. Despite previous evaluation studies of the Midwest CPC program showing evidence of an increase in parent involvement by end of preschool (Reynolds et al., 2016, Richardson et al., 2017), the impact several years later was unknown. Given the concern of positive preschool impact “fading out” or “dropping off” in other preschool evaluation studies, our findings show the promising prospect of sustaining gains. We attribute this high parent involvement in early elementary grades to the CPC program’s emphasis on active parent engagement in child development. As the longitudinal path analysis of early parent involvement on student achievement from the CLS suggests (Hayakawa et al., 2013), early parent involvement influence kindergarten and first-grade academic motivation of children, which in turn encourages parents to continue active engagement in child development throughout later grades. We hypothesize that our findings are a result of a similar cyclical process shown from the CLS.

Another highlight of this study is the way we measured parent involvement as a composite measure utilizing information from multiple sources. As our study was implemented in public school districts across numerous sites, inconsistency in data collection was inevitable. Despite the implementation effort to be consistent across sites, there were difficulties in collecting the same parent involvement information. Moreover, with student mobility and study attrition over time, not all data collection was available over the implementation years. Our study shows how a composite measure can be created using multiple data sources. This way of incorporating multiple data sources to account for missing data can help researchers to overcome implementation barriers and further help accurately evaluate the program's impact.

This study evaluates the impact of the CPC program not only in Chicago but also in St. Paul district as well as both districts combined. Our findings show that the positive CPC program's impact on second-grade parent involvement is mainly driven by the Chicago district. This may be because the Chicago district had a longer history of offering a CPC program with an emphasis on parent involvement to both schools and parents. In St. Paul, however, the Child-Parent Center program was new to the district and there may have been a ‘startup cost’ where it takes time for schools and parents to develop expectations of parent involvement in school settings. One reason for the lack of strong significance in St. Paul may be the small amount of variation in the parent involvement measure in second grade for St. Paul district, where the majority of parents were categorized as participating in school less than once a month.

### 6.1 Relationship to earlier results on Child-Parent Centers

Previous research has reported on the link between participation in the Child-Parent Centers from an earlier cohort. How do our findings compare to the Chicago Longitudinal Study (CLS) of the CPC program in the 1980s? Based on teacher ratings of parental involvement in school, Reynolds (1995) reported an effect size of 0.32 SD for CPC preschool on teacher ratings of parent involvement in school in second grade. This estimated effect was stable over time as effect sizes for first and third grades were also sizable (0.51 and 0.44 SD, respectively). The findings in the current study show a smaller effect size, though several differences between the two CPC studies are noteworthy. First, the measure in the current study is a composite of teacher and parent ratings and is likely to capture a wider range of school involvement practices. Second, whereas parent involvement in school was the major component of family support services in the 1980s CPC program, it was more balanced in priority among home and community involvement in the present new-generation program.

The third difference between the two CPC studies is that the participants in the current study are racially and ethnically more diverse than in the CLS. Over 90% of the CLS sample was black whereas it is less than 50% in the present study with much larger percentages of Latino and Asian families. These demographic differences may account for some of the differences in effect size and the fact that the implementation of the family support component was the first year of a large school reform expansion. Moreover, as noted in the introduction, parent involvement has also been identified as a major mechanism of change that accounts for the long-term effects of the program on educational attainment, income, and other measures of well-being (Reynolds & Ou, 2011). This suggests that, despite a smaller effect size reported in the current study, parental involvement is likely to contribute to cumulative advantages that lead to positive well-being in adolescence and beyond.

Finally, there are two key differences in the study design comparing the older and newer cohorts. In the Chicago Longitudinal Study of children born around 1980, the comparison group consisted of children who generally lacked preschool participation experience. In the newer cohort study, the comparison group children attended publicly funded non-CPC preschools offered in high-poverty schools (in St. Paul) and offered universally (in Chicago). Second, the outcome regressions reported in this research include a pre-test available for both treatment and control groups that was not part of the study design for the older study.

### 6.2 Limitations and future directions

There are limitations in our study that need further research. First, while home support for learning is more emphasized in the Midwest expansion of the CPC program as opposed to the original CPC program, we did not assess home parental involvement. Our measure of parent involvement focuses only on participation in the school setting because the main purpose of the CPC program and the Midwest expansion was to strengthen the family-school relationship. Although the focus of this study is on parent involvement in school, we acknowledge the importance and the need of examining the program's impact on other parent involvement measures outside of the school setting. For example, examining home involvement and parent expectations in the parent involvement measure could yield larger program effects than reported in earlier studies. Jeynes (2010) suggests that educators and early childhood program developers should recognize the importance of more subtle forms of parent involvement such as expectations for success rather than focusing on easier to measure or monitor forms such as homework checking or attendance at school events. Hayakawa et al. (2016) also emphasize the role of parent expectations as a form of parent involvement that may enhance students’ commitment and motivation for school. Which of the two forms of involvement is more important for student achievement is also contested in the literature. While Lee and Bowen (2006) found that parent involvement at school showed the strongest associations with students’ achievement, Barger et al. (2019) suggest that school involvement perhaps has a smaller impact than cognitively stimulating their children.

Next, our evaluation of the MCPC program on second-grade parent involvement is limited to two districts, Chicago and St. Paul. The MCPC program was implemented in two more districts of Illinois - Evanston, and Normal. These two districts did not have comparable comparison sites to accurately evaluate the program impact, which is why they were excluded from our initial analysis. Future work is needed to evaluate the impact of the program on these districts to capture the overall program impact holistically. This will help us get a better understanding of the impact of the MCPC program in school districts outside of Chicago that do not have a history of the original CPC program.

## Funding

Preparation of this manuscript was supported by the U. S. Department of Education Office of Innovation (U411B110098), 10.13039/100000071National Institute of Child Health and Human Development (No. HD034294), and the 10.13039/100000865Bill & Melinda Gates Foundation (No. OPP1173152). The funders had no role in the manuscript preparation and decision to submit for publication.

## CRediT authorship contribution statement

**Nishank Varshney:** Conceptualization, Data curation. **Sangyoo Lee:** Conceptualization, Data curation. **Judy A. Temple:** Conceptualization, Methodology, Funding acquisition. **Arthur J. Reynolds:** Conceptualization, Methodology, Funding acquisition.

## Declaration of Competing Interest

The authors declare that they have no known competing financial interests or personal relationships that could have appeared to influence the work reported in this paper.

## Figures and Tables

**Table:** 

	More than once a week	Once a week	2–3 times a month	Once a month	Less than once a month	Never
1. Participate in any school or center activities						
2. Helps you with assignments or activities						
3. Meet with you or other teachers						
4. Discuss child’s progress						
5. Go on field trips						
6. Volunteer in the school or classroom						
7. Attend school events						
8. Attend workshops						
9. Receive home visits						
10. Attend parent-teacher conferences						
11. Attend meetings of the parent-teacher association or another group						
12. Plan school trips						
13. Plan classroom activities						

**Table:** 

Variable	Chicago		St. Paul	
	CPC	Comparison	CPC	Comparison
Female	1724	906	297	201
Race/Ethnicity	1724	906	298	201
Special education status	1724	905	297	201
Enrolled as three-year-old	1724	906	n/a	n/a
Child eligible for free or reduced lunch	1724	906	297	201
Mother completed high school	1138	317	140	65
Single parent family status	1176	319	148	67
Mother employed full- or part-time	1153	315	146	66
Home language other than English	1697	850	212	115
Fall preschool score on Literacy	948	428	282	187
Fall preschool score on Math	987	444	n/a	n/a
Fall preschool Socioemot. devel	948	371	n/a	n/a
Fall preschool score (total), mean	767	320	n/a	n/a
Note: Data on child and family risk variables were collected from school administrative records and parent surveys.				

**Table:** 

Variable	Regression Model	IPW Model
CPC Program Participation	Yes	Yes
Standardized Fall Baseline Test Score	Yes	Yes
Single parent family status	Yes	Yes
Mother employed full- or part-time	Yes	Yes
Mother completed high school	Yes	Yes
Child eligible for free or reduced lunch	Yes	Yes
Household income within 185% of federal poverty	Yes	Yes
Family moved in last 12 months	Yes	Yes
Family receives any government assistance	Yes	Yes
Race (dummy variables)	Yes	Yes
Female	Yes	Yes
Family belongs to immigrant/refugee group	Yes	Yes
Special education status	Yes	Yes
Household native language not English	Yes	Yes
District	Yes	Yes
Mother's age at childbirth	No	Yes
Parents satisfied with the children's school	No	Yes
Child low birth weight (<2500 g)	No	Yes
Whether the family felt food insecure in last six months	No	Yes
Family had more than four kids	No	Yes
Family lived in safe neighborhood	No	Yes
Note: Data on these variables were collected from school administrative records and parent surveys. Both models also include a set of binary variables that indicate whether missing data was imputed for that explanatory variable.		

**Table D1:** Implementation Fidelity Scores for Preschool Year (SY12-13) – Chicago.

Element	Mean	Standard Deviation	Minimum	Maximum
Effective Learning Experiences	3.6	0.6	3.0	5.0
Collaborative Leadership Team	4.0	1.0	2.0	5.0
Aligned Curriculum	3.6	0.6	3.0	5.0
Parent Involvement	3.9	1.3	1.0	5.0
Professional Development	3.4	1.1	1.7	5.0
Continuity and Stability	4.3	0.7	3.0	5.0
Overall	3.8	0.5	3.3	4.8

**Table D2:** Implementation Fidelity Scores for Preschool Year (SY12-13) – St. Paul.

Element	Mean	Standard Deviation	Minimum	Maximum
Effective Learning Experiences	5.0	0.0	5.0	5.0
Collaborative Leadership Team	5.0	0.0	5.0	5.0
Aligned Curriculum	5.0	0.0	5.0	5.0
Parent Involvement	3.5	1.0	2.0	5.0
Professional Development	4.1	0.3	3.7	4.3
Continuity and Stability	4.0	0.0	4.0	4.0
Overall	4.4	0.2	4.2	4.7

## Appendix A

Year 4 (2nd grade) teacher checklist

About how often does {student}’s parent(s) participate in the following?

## Appendix B

Sample size for child and family risk variables

## Appendix C

Variables included in regression and inverse probability weighting (IPW) models

## Appendix D

See Table D1, Table D2.
